# A summer of phobias: media discourses on ‘radical’ acts of dissent against ‘mass tourism’ in Barcelona

**DOI:** 10.12688/openreseurope.13253.1

**Published:** 2021-06-10

**Authors:** Alexander Araya López

**Affiliations:** 1 Department of Linguistics and Comparative Cultural Studies, Ca' Foscari University of Venice, Venezia, Venezia, 30123, Italy

**Keywords:** social movements, journalism, radical politics, public sphere, cities, tourism

## Abstract

In the summer of 2017, the young group *Arran* coordinated a series of protests in Barcelona and other Spanish cities to denounce the negative effects of global mass tourism. These acts of dissent fueled a heated public debate in both Spanish and international press, mainly due to the ‘radical’ tactics employed by the demonstrators. Following the narratives about these protest acts across a diversity of media outlets, this article identifies the complex power struggles between the different actors involved in the discussion on the benefits and externalities of global mass tourism, offering an extensive analysis of the political uses of the term ‘turismofobia’ (tourismphobia) and a revisited interpretation of the notion of the ‘protest paradigm’. This qualitative analysis was based on more than 700 media texts (including news articles, op eds and editorials) collected through the database Factiva, from January 2014 to December 2017.

## Introduction

Social movements rarely rely on a single strategy to elevate their claims to the public sphere. Lobbying and signature collection campaigns, performances, awareness campaigns, mass demonstrations, strikes, press releases, documentaries, websites and accounts on social media and other physical manifestations of dissent (i.e. graffiti, banners, posters, public art, etc.) could be used to keep pressure on both local authorities and the (global/local) media, otherwise they risk losing momentum, and their cause could disappear from the public debate. Habermas (1990 [1962]); Habermas (1996) has extensively explained the importance of this ‘publicity’ for the functioning of any deliberative democracy, which allows social actors to ‘secure’ (to some extent) their right to participate in opinion- and will-formation and decision-making processes. Given that participation in the public debate is far from ideal, disenfranchised groups might need to appeal to performative strategies to capture media attention ( Avritzer & Costa, 2004) – including acts of civil/democratic disobedience ( Celikates, 2016; Markovits, 2005) – oftentimes constituting subaltern counterpublics ( Fraser, 1990; Warner, 2002), thus offering alternative interpretations of a given subject and enriching the ‘rational discussion’ of public matters.

This paper analyzes the media discourses and narratives regarding a series of acts of protest against the negative effects of mass tourism in Barcelona. Social movements in this city have been denouncing the various externalities of global mass tourism for years and there is a broad corpus of academic studies that address these ‘protests’ and campaigns: Arias-Sans & Russo (2017) discussed the protest acts against the enclosure of Park Güell; Mansilla & Milano (2018) documented the actions of the collective *EnsPlantem* against the ‘touristification’ of Poblenou; Nofre *et al*. (2018) examined the impact of tourism gentrification linked to the emergence of a nightlife scene in La Barceloneta neighborhood; Hughes (2018) studied the emergence of anti-tourism protests and provided a short analysis of the political debate related to the protest acts coordinated by the young collective *Arran*; while Bruttomesso (2018) has provided a detailed review of the playful protests by the collective *Fem Plaça*, which aims at reclaiming public spaces in Barcelona. In short, there are a variety of social movements denouncing the side effects of mass tourism in the city, referencing problematic outcomes that include but are not limited to the lack of social housing and the emergence of tourist rentals (mostly Airbnb, but also Booking.com, Expedia, etc.), the ‘inappropriate’ behavior of tourists ( *incivismo*), the externalities of the cruise industry, the aircraft noise and air pollution associated with the El Prat airport, overcrowding and privatization of public spaces, or the ‘touristification’ of living spaces.

As part of broader research on media discourses including other European cities affected by global mass tourism (namely Venice and Amsterdam), this paper focusses on a series of protest acts that took place in Barcelona in the summer of 2017, mainly involving the young group *Arran*, a left-wing collective linked to the local party CUP (Popular Unity Candidacy) ^1^, but also involving other groups and social movements from across Spain. These events led to a complex public debate spanning local and international media, which involved politicians (at the local and national level), business lobbies, scholars, citizens and tourists. Although Arran does not represent the diverse movements ‘fighting’ global mass tourism in Barcelona, the collective has gained notoriety for employing tactics and strategies deemed more ‘transgressive’ than those selected by other groups. It was precisely their use of these ‘radical’ politics that granted Arran (and its associated groups) a sort of ‘singularity’ within local and international media, partially because of the ‘spectacular nature’ of their actions, but also on account of their acts of dissent fitting the idea of *‘turismofobia’* (subsequently *tourismphobia*), a highly ‘biased’ term that gained popularity among politicians, journalists and some stakeholders of the tourism industry.

Although this paper refers to the side effects of global mass tourism, the main discussion presented here is about ‘radical’ protest acts and their representation as media discourses. There are a plethora of studies and specialized reports that provide a valuable analysis of the negative outcomes of the global tourism industry, which includes a volume examining tourism in diverse urban settings, edited by Colomb & Novy (2017); a compilation comparing several cities in Latin America affected by mass tourism with other European cities of the Iberian Peninsula, by Milano & Mansilla (2018); a two-volume report by United Nations World Tourism Organization ( UNWTO, 2018) entitled Overtourism’? – Understanding and Managing Urban Tourism Growth beyond Perceptions, Executive Summary; which includes summarized chapters for numerous ‘travel destinations’; or the volume edited by Cañada & Murray (2019), with several critical approaches to mass tourism, including perspectives from feminism and environmentalism. All these studies provide verifiable information regarding the (local) impact of (global) mass tourism, at various levels such as economic, physical, socio-cultural and psychological ( Colomb & Novy, 2017). 

This article has been structured in five sections. Firstly, a concise review of media studies theory referring to how ‘the news’ are constructed and consumed, including an introduction to the notion of the *protest paradigm* (and *marginalization devices*). Secondly, a detailed explanation of the methodology that highlights the scope of the analysis. Thirdly, the protest acts organized by Arran (and other demonstrations that gained media attention in the summer of 2017) are scrutinized, followed by a discussion of the main discourses and narratives identified in these media texts, organized in thematic subsections. Fourthly, a short reference to an interview with a spokesperson from the collective Arran has been included, in order to balance the information presented by both local and international media. The last section presents the main conclusions and offers some suggestions for improving the quality of media texts reporting on acts of dissent.

### What is ‘the news’? Newsworthiness and the consumption of media

Media studies, according to Martini (2000) could be oriented towards the processes involving the producers of ‘news’, the published content (i.e. texts, images, colors, diagrams, etc.), or the audience/readership and their ways of consuming news. This paper belongs to the second category, thus deeming journalistic practices like the selection of sources and the individual behaviors and preferences of consumers outside of the scope of this study (although these processes are somehow implicit in the published media texts). Similarly, the complex dynamics between different media outlets (for instance, competition between different newspapers) and the political agendas of each media outlet (and the global corporations that own them) are not considered here. Moreover, newspapers and other media outlets oftentimes have difficult relationships with other powerful actors in a given society, for example, considering their dependence on the revenue obtained through advertising or their dependence on information provided by local authorities (in the sense that media would be forced to ‘restrain themselves’ to perpetuate their access to power or information) ( Martini, 2000: 62–68). Lavy *et al*. (2016) have pointed out the need to understand the inherent bias in media as a way to disentangle complex social power dynamics, considering that media outlets tend to promote growth-oriented development.

The process of producing ‘news’ is highly complex yet characterized by some fundamental notions. The *‘*news *’* is a construction, meaning that a ‘factual event’ that has taken place in society has been considered ‘newsworthy *’* by someone ( Bednarek & Caple, 2017; Martini, 2000). This decision is made by editors and journalists based on several criteria: novelty, quantity of people involved, participation of important figures or personalities, potential to create other secondary news, or the geographical proximity of the events ( Martini, 2000: 89–95). Many events – including ‘peaceful’ protests against tourism – are perceived to be uninteresting or not dramatic enough to become ‘news’ (or they might receive limited coverage). The production of news is dependent on other more practical concerns such as the allocation of resources, including time constraints to produce a given reportage, the number of available journalists, or the unused space on the paper on the printed versions. Moreover, the quality of a ‘news article’ could be affected by other ‘assumptions’, as Martini (2000: 40) calls them, referring to the shortcuts that are taken in relation to readers/audiences, such as assuming readership awareness of particular details or historical backgrounds. The role played by news agencies (i.e. *Associated Press, Reuters, Deutsche Presse Agentur*) in providing information to both local and global media outlets should not be ignored in relation to news production processes, given that it might explain the similarities between ‘news articles’ published across different media outlets, including some extreme cases of downright ‘copy-pasted’ publications. This reliance on news agencies could negatively affect the quality of the articles in various ways, for example by encouraging oversimplification or homogenization. 

In relation to the consumption of media, the hypodermic needle model of acritical consumption of media contents, in which the audiences were considered passive and acritical, has been long abandoned. According to Hall *et al*. (1980), the readers could negotiate the contents published by media or they can accept them or reject them altogether. This process might vary according to several structural determinants such as ‘class’ or ‘education’, but it could also be shaped by personal experiences (for this paper, for example, citizens of Spanish cities affected by mass tourism might be more sympathetic towards Arran’s actions than those who are relatively protected from the various impacts of the industry). In the digital age, the interaction that readers and audiences have with media contexts is extremely complex, as they can express their negotiation, acceptance or rejection of the ‘news’ in the form of comments, likes/hearts, retweets, etc. (while in the past, this process offered more limited options such as letters or appeals to the ombudsman, if any). Moreover, social movements denouncing the negative effects of mass tourism frequently fall under the definition of *prosumers* ( Ritzer & Jurgenson, 2010) because they are able to consume the media while simultaneously producing new content, either to oppose a given narrative or to complete it with their own information, experiences or interpretations. Social movements frequently share the ‘news’ on their social media channels, either to support their cause or to challenge the statements made by the media, local and national authorities, private businesses and corporations, etc. These digital (re)actions and the ensuing online debate about the media texts analyzed here have not been investigated.

### The protest paradigm

The *protest paradigm* refers to a series of journalistic practices that present a biased narrative about both protests and protesters. Following McLeod (2007), one of the main concerns about the uses of the protest paradigm is that it obstructs more constructive forms of news coverage, while it also prevents multi-perspective approaches. In his view, media tend to emphasize *news frames* that focus on conflict (i.e. the crime story, riot, carnival, etc.) instead of choosing a frame based on public debate. The protest paradigm shows a preference for official sources, considering that these powerful individuals add value to the news article in the form of prestige, while reinforcing the idea of objectivity (see also Martini, 2000). Furthermore, these types of media stories on protest tend to comment on the appearance and behavior of protesters and might overemphasize ‘violent’ events involving violations of the law (i.e. civil/democratic disobedience). The paradigm also ‘constructs’ or refers to public opinion in the form of offering non-verifiable generalizations, or by relying on bystander portrayals of the events. McLeod (2007) argues against both the delegitimization and demonization of protests, with the former being a process in which media texts fail to provide the necessary context to understand the actions of the protesters while the latter consists of a sort of ‘moral panic’ (folk devils) created by exaggerating threats, risks and harms.

Dardis (2006) has classified a series of *marginalization devices* frequently used by media to cover protest acts, hence portraying them negatively. These mechanisms include references to general lawlessness, confrontation with the police, freak show, idiots at large, carnival, actual statistics, generalizations, witness accounts, official sources, protests as treason, protest as anarchy, protests as anti-troops (for anti-war protests mainly), inclusion of counterdemonstrations, and historical comparisons. These marginalization devices have been identified in the media texts about a diversity of protests, from the U.S. press coverage of the Iraq war ( Dardis, 2006) to other acts of dissent in China, India and Brazil ( Shahin *et al*., 2016). Nonetheless, these marginalization devices rarely appear in a ‘pure’ form in the news articles, and there seems to be a flexibility (which might vary according to journalist or media outlet) regarding the ‘framing’ of acts of dissent.

Both the protest paradigm (including its delegitimization and demonization of protests) and several marginalization devices summarized here were found in the news articles, editorials and op eds discussing the ‘radical’ acts of dissent orchestrated by Arran in Barcelona and Palma de Mallorca in the summer of 2017, although in a ‘softer’ version, as it is discussed in the following pages. 

## Methods

With the objective of tracking the media discourses about the acts of dissent against ‘mass tourism’ in Venice, Amsterdam and Barcelona, a series of texts in both local and global media outlets were collected through the aggregation database Factiva, for a total of 723 valid articles ^2^ published between January 1 ^st^, 2014 and December 31 ^st^, 2017 ^3^. The search for these media texts was based on a combination of keywords, including: overtourism, *tourismphobia/turismofobia*, mass tourism, social movements, protest, right to the city, etc. The texts were collected in several languages, including English, German, Spanish/Catalan, Portuguese, and Italian. The keywords included the three city-cases defined for this research, with specific variations due to language (for example: Venice, Venecia, Venezia, Venedig).

Following the methodology proposed by Tonkiss (2004), the discourse analysis presented here is based in three different processes: a) Identifying key themes and arguments, b) looking for variation in the text, and c) paying attention to silences (in order to identify what is excluded by omission or any gaps in the media coverage). After a review of current literature on mass tourism and social movements, more than 50 codes were created to arrange and retrieve information, using the computer-assisted qualitative data analysis software (CAQDAS) *NVivo*. The frequency of certain themes or sources was not the main goal of this research, and therefore these codes were not used for quantitative analysis. In this sense, several codes could be used to tag the same segment of a particular text. The following table ( Table 1) includes a short selection of these codes.

**Table 1.** Codes for qualitative analysis of media texts (excerpt).

Code	Description
HOTEL	Hotel industry (i.e., hostels, guest houses, bed and breakfasts, etc.), particularly the construction/banning of new hotels. NOT for tourist rentals. It includes working conditions of personnel, seasonality, etc.
TRENT	Tourist rentals that are NOT hotels (including summer apartments) independently of the platform in which they are advertised (Airbnb, Booking.com, Expedia, etc.)
LOGOV	References to the local government and politicians (includes regional government, if necessary).
NAGOV	References to the national government and major political parties
EUGOV	References to the European government (European Commission, Europe as administrative unit, etc.)
RIGHT	Any description of demonstrations, rallies, public occupation of spaces, marches and other type of collective or individual expression of dissent, in support or against global mass tourism. It includes techniques not related to the occupation of public spaces such as collection of signatures, lobbying and petitions (such as online campaigns).
RADPO	A sub-code of RIGHT in relation to the use of ‘radical politics’, such as violent or non-violent manifestations of dissent (i.e., performances, ‘attacks’, graffiti and stickers, squatting, destruction of public property, etc.)
VSTRS	For description of tourists as visitors of a destination, collectively or individually. Tourist as plagues, as ignorant, as masses, as poor, etc. It covers any comparisons in the narrative ‘tourists versus locals’, which describe different consumption patterns and market ‘needs’, clashing schedules, diverse rhythms and ways to interact in the public space, etc.
POMIL	In relation to the police and military (including civil police and private security). Any allusions to international terrorism are included in this code.
GRWTH	References on the role of tourism in fostering economic growth, for example: employment data, revenues, both public and private investments, GDP, etc.

Source: Author

In terms of the scope of this research, and particularly in relation to Factiva, it is important to mention that this database aggregates news from a diversity of media outlets, including news agencies such as *Deutsche Presse-Agentur* or *Associated Press*, local media such as the *Diario de Mallorca* and global outlets like *The Telegraph* or *The Guardian*. Therefore, the findings presented here follow the ‘global debate’ about the subject of mass tourism and dissent, and do not represent the political orientation of a single newspaper or media outlet. Additionally, Factiva collects the texts without systematic access to the images, diagrams, maps and other visual resources, significantly limiting the discourse analysis to the written word. In order to include this visual data, some selected articles deemed particularly informative were correspondingly checked on the official website of the news outlet, but this task was not possible for all articles (considering both pay-walls and printed texts not available in digital archives).

Factiva identifies these media articles according to the keywords given to the search engine, and the algorithm used for this information retrieval is not known or controlled by the researcher, with the results including both unrelated articles (‘false positives’) and the systematic collection of similar articles (duplicates). When these articles were similar enough, they were excluded ^4^. Under no circumstances does this mean that all the articles of a given newspaper or media outlet were collected, and if a second researcher uses different keywords for the search engine in Factiva, the database would provide different results (especially when working with a variety of languages). Although there are some clear limitations, the analysis of the texts and the information collected through Factiva, combined with the advantages of the use of CAQDAS NVivo, has eased the process of distinguishing common themes and tracking variation in the media narratives across a diversity of media outlets.

Another methodological decision to take into consideration regarding the media texts examined here refers to the selection of valid articles. This research was originally proposed to analyze the media narratives about acts of dissent concerning mass tourism in Venice, Amsterdam and Barcelona. However, for the Barcelona city-case and in relation with the issue of *tourismphobia*, the events extended both geographically and narratively, and the discussion included other Spanish cities such as Palma de Mallorca, Valencia, Madrid or Bilbao. Indeed, Arran and other associated groups protested in most of these cities, and media followed these ‘events’. Rather than excluding these articles as off-topic, the analysis was expanded to integrate this valuable information, which made visible some specific issues about ‘Spain’ as a tourist destination that would have been missed in a study focusing exclusively on Barcelona.

### Ethics

The RIGHTS UP project has safeguarded the conditions of privacy, anonymity, right to withdrawal, ‘dual use’, ‘incidental findings’, proportionality and other legal and ethical principles that secure the well-being of the individual research subjects, while keeping all personal data safe from unforeseen, unintended and malevolent use. Both the public information collected from media outlets and social networks (for example, Twitter) and the additional in-depth interviews with key informants included in this publication followed the principle of ‘no harm’. In the case of the four in-depth interviews mentioned in this article ^5^, a written consent form was discussed and signed with the human participants previous to any recording. The RIGHTS UP project submitted its ethics guidelines to the respective Ethics Committee at Ca' Foscari, University of Venice at the beginning of the project, and the approval was received on July 2 ^nd^, 2018.

## Results

### The protests against mass tourism: a summary of events

Considering the period of study for the collection of news articles, the first ‘event’ identified in the media texts was a series of protests that took place in La Barceloneta in the summer of 2014. It is important to mention that the process of converting Barcelona into a ‘tourist city’ could be tracked back at least three decades to its nomination to host the 1992 Summer Olympic Games (see: Hughes, 2018; Milano & Mansilla, 2018; Russo & Scarnato, 2018; Smith, 2005). Although there were protests before 2014, including for example of the many acts of dissent against the enclosure of the renowned Park Güell and the instauration of a paid ticket for visitors starting in 2013 ( Arias-Sans & Russo, 2017), it was the incident with three Italian tourists who wandered naked around La Barceloneta that became a turning point in the ‘fight’ against ‘mass tourism’ ( Couzens, 2014). For years, the ‘unruly’ behavior of tourists in La Barceloneta (particularly those who are labelled as low-cost, party tourists) was associated to the popularity of tourist rentals, in specific Airbnb, which was becoming a serious issue in Barcelona at the time. Indeed, it was after this series of protest acts in 2014 that the local government started to regulate the tourist rental platforms, aiming at reducing both the complaints about the ‘disruptive’ behavior of tourists and to contain the emergence of illegal accommodation. For many activists and neighbors in ‘touristified’ Barcelona, these actions (or promises) of the local government were insufficient.

This paper focuses on a series of protest acts that occurred three years later, in the summer of 2017. Since June 2015, Mayor Ada Colau from the party *Barcelona en Comú* was in charge of the local government of Barcelona after a political campaign that explicitly addressed the shared concerns about mass tourism in the city. Although some steps regarding the management of tourism were taken (for example, a ban against new hotels in early 2017 ^6^ and heavy fines against digital platforms for advertising unauthorized tourists rentals ( Hughes, 2018)), the popular dissatisfaction with mass tourism was commonplace.

The media texts reporting the protest acts by Arran did not necessarily follow a chronological order and it could be said that the so-defined ‘attack’ against a sightseeing bus coordinated by four members of the group near Camp Nou in Barcelona was the single event that captured the most media attention in the national and European debate about the issue of ‘overtourism’ and ‘tourismphobia’ in 2017. A second ‘attack’ organized by Arran (reported later by the media but which indeed took place several days before the first incident) consisted of a group of protesters allegedly scaring tourists away at a restaurant in Palma de Mallorca. A possible explanation for this discrepancy might be due to the ‘moment’ in which these events were made public by Arran on their official media channels. Nonetheless, these two events were extensively discussed in local and international media, including several references in British and German publications, which constitute the main two ‘suppliers’ of tourists for the Spanish market.

The following list chronologically identifies the events that took place in the summer of 2017:

- **July 22 ^nd^, 2017 – Palma de Mallorca:**
  Arran organizes a protest with banners at a restaurant in Moll Vell, in Palma de Mallorca. The group allegedly scared the customers with red flares and confetti. The banners included messages such as “Tourism kills Mallorca” and “The class struggle is being fought here”. A video of the action was published on the Twitter account *Arran* on August 1st, 2017. The tweet includes the legend: “We stop the mass tourism that destroys #Mallorca, which condemns the working class of the Catalan Countries to misery! #LaClau (key emoji)”. The video is musicalized with the song ‘ *Not welcome’* by *Lágrimas de Sangre* and the lyrics include explicit references against tourists in Barcelona and other Spanish cities. Months later, the organizer of the protests was fined 1200 EUR as reported in the Spanish news: 900 EUR for the use of pyrotechnics without authorization and 300 EUR for organizing an unauthorized demonstration in a public space ( El Mundo, 2017).

- **July 27 ^th^, 2017 – Barcelona**:
  Four members of Arran stop a sightseeing bus and spray a message on the windshield, declaring: “Tourism kills the neighborhoods”. Several media articles report that the protesters slashed the tires of the vehicle, but other media such as *Diario de Mallorca* deny this fact ^7^. A British tourist is recurrently quoted by several media outlets, testifying that he believed the bus “was being attacked by terrorists” ^8^. The damages were estimated at 1,849.24 EUR (including material damages and the interruption of the service). A video of the ‘attack’ was shared on the Twitter account *Arran* on July 30 ^th^, 2017. The footage shows overcrowded streets in Barcelona, standardized restaurant menus, cruise ships and go cars. The video is musicalized with the song *‘Tot Explota Pel Cap o Per la Pota’* by *Inadaptats*. The tweet also says: “Mass tourism kills neighborhoods, destroys territory and condemns the working class to misery. #autodefensa (fist emoji)”

- **July 31 ^st^, 2017 – Barcelona:**

*Arran* slashes the tires of rental bikes in the Poblenou district in Barcelona and a video is uploaded on the Twitter account *Arran del Poblenou* with the message: “We are fed up with the occupation by tourist companies of the public space of the neighborhood, ACT! JOIN THE COMBAT (raised fist emoji)”. The video is musicalized with the song *‘Ciutat Morta’* by *Kop*. Media texts repeatedly referred to this protest act as ‘vandalism’.

- **August 6 ^th^, 2017 – Online statement:**

A press release is published by Arran on their website, denouncing the current management of tourism and demanding five central actions: a) to deny licenses for hotels and tourism businesses, b) to improve work conditions in the sector, c) to increase the taxes for businesses in the tourism industry, d) to ban with immediate effect all tourist rentals (with a specific reference to Airbnb), and e) to expropriate the main tourist assets (including the hotel Vela in Barcelona and theme parks such as Port Aventura). A few Spanish media outlets reported this ‘manifesto’, including Spanish newspaper *La Vanguardia* and the *Huffington Post Spain*.

- **August 9 ^th^, 2017 – Bilbao:**

The group *Ernai* posts a video on Twitter depicting a couple of people spraying political messages and throwing red paint against the external walls of the Agencia Vasca de Turismo. The video is musicalized with the song *‘Gnars Attacks’* by *I See Stars*. Another tweet suggests that this ‘attack’ was motivated by a request made by local authorities to cancel a demonstration planned for August 17 ^th^, 2017.

- **August 9 ^th^, 2017 – Palma de Mallorca:**

*Endavant* Mallorca, *Arran* Palma and the environmentalist group *GOB* (Balearic Group of Ornithology and Defence of Nature) posted 1000 stickers on rental cars as a way to protest against mass tourism. A couple of tweets explained that there are 100,000 rental cars in Mallorca, and that #AquestCotxeSobra (This car is too much).

- **August 9 ^th^, 2017 – Donostia (San Sebastian):**

A group of disguised protesters wearing colorful wigs linked to the group *Ernai* holds banners with messages against tourism and surrounds the small tourist train *Txu Txu* in Donostia (Euskadi). The video is published on the YouTube channel *Ernai Donostia* and via Twitter, showing protesters throwing confetti, using flares and dancing. The popular song *‘Macarena’* by *Los del Río* is used as a background. Some tourists in the train are seen filming and photographing the event.

- **August 12 ^th^, 2017 – Barcelona:**

Neighbors of La Barceloneta symbolically occupy the beach, carrying banners and forming a human chain alongside the shore. The banners included messages such as ‘We don’t want tourist (sic) in our buildings! This is not a beach resort’ and ‘For the abolition of holiday rentals’. The protesters wore yellow t-shirts with the slogan ‘Barceloneta is not for sale’ written in Catalan. The action was part of a city-wide campaign #CapMésEstiuComAquest (No more summers like this one) coordinated by the *Association of Neighborhoods for Sustainable Tourism (ABTS)* and the local group *La Barceloneta diu prou* (La Barceloneta says enough). Several news articles describing this protest act emphasized the contrast between tourists napping on the beach and locals demonstrating ‘angrily’. While this protest was not organized by Arran, the act was read as another manifestation of ‘tourismphobia’.

During this summer other minor events were included in the media coverage: a call to protest for animal rights (particularly against the use of horses in carriages) organized by the group *Prou Tracció A Sang*, a ‘sabotage’ of recently-installed hammocks in Canarias (which could be attributed to common criminality, as reported in a news article), a ‘photocall’ near Vallcarca in Barcelona with a legend stating ‘I’m a mindless consumerist tourist’, political statements and popular concerts against parties in luxury yachts in Ibiza ( Colmenero, 2017), etc. These minor actions were similarly categorized as expressions of ‘tourismphobia’. Moreover, there were news articles referring to communities allegedly ‘free of tourismphobia’, pointing out that these places were open to welcome tourists and did not experience any protest acts, such as the Spanish capital Madrid.

### The media discourses: ‘tourismphobia’, politics, branding and the right to protest

Through the analysis of these media texts (news articles, op eds, editorials, letters from readers), several discourses were identified, which are explained in the following subsections. It is important to clarify that these narratives do not appear as isolated themes and in practice are extremely intertwined. 

***Tourismphobia and overtourism*.** At first glance, ‘tourismphobia’ seems to have a singular meaning in these media texts: the local discontent associated to the massive presence of tourists. There are two significant aspects that must be addressed in relation to this concept: first, the newspapers and journalists seem to use this term interchangeably to represent both the ‘protests’ or demonstrations and the ‘sentiment’ that is growing within the ‘affected’ communities. Second, ‘tourismphobia’ is conceptually linked to mass tourism, to a certain idea of overcrowding (similar to the idea of ‘overtourism’). However, these are both partial interpretations of the phenomenon.

By equating tourismphobia to acts of dissent, the media narratives lose track of the diversity of protest strategies and ‘ideological’ positions among those who are critical of the global tourism industry. For example, the discussions about the causes of ‘overtourism’, the strategies to communicate the impact of these ‘externalities’ to the public or the potential solutions are oftentimes long and marked by disagreements within the same ‘antagonist group’. While there is not enough evidence to suggest that these media narratives comply with the ‘protest paradigm’ mentioned above, there are some paragraphs and quotations from powerful sources that clearly stigmatize protest acts and overemphasize the spectacular nature of these demonstrations. Particularly when referring the acts of dissent organized by Arran, several media reported the ‘loud and violent’ music accompanying the videos, the presence of ‘disguised’ protesters and the ‘angry’ attitude of the participants. The texts repeatedly described the ‘vandalism’ of these protest acts, by emphasizing the clash with local authorities and the police (mostly in the form of official reports and public condemnation from legitimate sources such as local/national politicians).

The usage of ‘tourismphobia’ as an equivalent for mass tourism and overcrowding likewise denotes a biased view of the problem. Although both play a role on aggravating some of the negative consequences of the global tourism industry, there are serious externalities that could not be solved by simply ‘reducing’ tourist numbers. In the case of ‘disruptive’ behaviors of tourists, the ‘few’ that engage in binge drinking or public sex easily overshadow the ‘majority’ of law-abiding, respectful tourists (especially during the ‘low season’ when less visitors are the norm) ^9^. Similarly, the overall decrease of affordable housing due to the transformation of these units into tourist rentals might be the result of the expectation of potential visitors, impacting the lives of locals significatively throughout the entire year, whether tourists are presently visiting the area or not.

The political uses of ‘tourismphobia’ have been noticed by several scholars who have questioned its validity within analytical settings ( Alcalde García *et al*., 2018; Huete & Mantecón, 2018; Milano *et al*., 2019; Zanardi, 2019). Even if ‘tourismphobia’ is a buzzword used by local politicians and journalists to ‘stigmatize’ protest acts, its meaning has not been uncontested: a reader ( Torres, 2017) characterized derelict places in need of public investment as a form of ‘tourismphobia’, and the term was mentioned in the headline of a news article criticizing the management of the Spanish government regarding the security staff strike at El Prat Airport in 2017 ( Diario Crítico, 2017a). Social movements, including the *Association of Neighborhoods for Tourism Degrowth* (formerly, Association of Neighborhoods for Sustainable Tourism) and the Arran group itself, have emphatically argued against the term ‘tourismphobia’, explaining that the target of their activism is the economic model that makes cities dependent on mass tourism and it does not imply a ‘phobia’ against visitors. This statement was often mentioned in the press.

Another political use of the concept was identified in relation to the hotel industry stakeholders, which often attributed the increasing ‘tourismphobia’ to the emergence of tourist rentals. This narrative functions as a strategy by the hotel businesses to separate themselves from the criticism directed toward the tourism industry, while attempting to sympathize with locals in their struggle against platforms such as Airbnb. It could be argued that the hotel industry stakeholders would benefit from any regulation imposed on Airbnb and similar digital platforms, and consequently it is ‘lucrative’ for them to frame tourist rentals as the main cause of ‘anti-tourism’ sentiments.

The public debate that took place in the summer of 2017 was a combination of several other ‘phobias’: in some news articles, ‘tourismphobia’ was paired with *xenophobia*, most noticeable in a declaration made by Jaume Collboni ( Benvenuty, 2017), a representative of the local government in an effort to censor ‘anti-tourism’ sentiments. Other political figures such as Carina Mejías ( *Ciutadans*) reportedly denounced the ‘criminalization of tourism’ ( Sust, 2017a). The term *guirifobia* appeared in a couple of texts as an equivalent to ‘tourismphobia’ ( Serna Andrés, 2017), although *guiri* is a pejorative slur generally used to refer to (wealthy) foreigners. Other phobias included the taxphobia ( *tasafobia* in Spanish), pointing out the resistance that local stakeholders in the tourism industry (particularly the hotel sector) show in relation to any taxes ( F.D.G. 2017). An article by Pau Morata (2017) speaks of excursionphobia ( *excursionofòbia)*, or the local discontent with day trippers whose contribution to the city is deemed ‘insufficient’, which was perceived by the author as a potential threat to ‘freedom of movement’. An article in *El Mundo* refers to neighborphobia ( *vecinofobia*), describing how the existing management of tourism impacts the lives of local populations ( Oms, 2017). Roger Pallarols, from the association of restaurateurs, used the term ‘institutional terracephobia’ ( *terrazofobia institutional*) in reference to the ‘excessive’ regulations and monitoring of the terraces ( Sust, 2017b) ^10^. Finally, the group Arran itself declared that their actions were not tourismphobia, but a fight against the ‘ *barricidio*’ (murdering of a neighborhood) ( Blanchar & Baquero, 2017). The concept of *barriofilia* also appears as a potential substitute to the narrative of ‘tourismphobia’.

The activism of Arran, which could be considered here as a form of ‘radical’ politics, was discursively associated to the notion of *‘kale borroka’*, a series of political and violent acts that constitute a form of urban guerrilla linked to the nationalist Basque movement ( Ramos, 2017). A few public figures such as Odón Elorza (a former mayor of San Sebastián) censured this comparison, even if he reportedly opposed the ‘attacks’ against tourism orchestrated by Arran ( Europapress, 2017). The ‘kale borroka’ narrative discursively linked the actions of Arran to the national struggles against domestic separatist terrorism (particularly in reference to ETA, Euskadi Ta Askatasuna). Pablo Echenique (from the political party *Podemos*) was quoted in an article denouncing the comparison between these protest acts and ETA (Huffington Post – Spain 2017). The ‘kale borroka’ narrative was also applied against the Spanish Prime Minister Mariano Rajoy and its *People’s Party* (PP), in a statement by Alfred Bosch (ERC – Republican Left of Catalonia), who characterized them ‘kale borroka with a tie’ in reference to the aforementioned strike crisis at El Prat Airport ( Vázquez, 2017).

***Local politics*.** The public debate on ‘tourismphobia’, overtourism and the acts of dissent was a ‘power struggle’ in which several local parties and tourism stakeholders expressed agreement/disagreement with each other, both concerning the management of tourism in Barcelona (or in other affected cities) and the ‘official’ response to the ‘radical’ politics employed by Arran. In this regard, the CUP was blamed for the actions of Arran, and the criticism included references to their ‘refusal to censor the violent attacks’ as well as to the political bargaining that the party was exerting apropos the upcoming 2017 Catalan Independence Referendum.

The complexity of this dimension of the debate between competing political parties will not be examined here, but it is important to mention that the criticism was predominantly directed against the Mayor of Barcelona Ada Colau and her team (explicitly, Agustí Colom, head of the Tourism Department). In short, Mayor Colau was blamed for exacerbating ‘tourismphobia’ (even during her candidacy campaign) and she was accused of being vague in her condemnation of the ‘violent’ acts of Arran ( Blanchar & Baquero, 2017). The disapproval was also extended to other prior decisions taken by the local government to ‘manage’ tourism, particularly the moratorium against new hotel developments.

This political debate included all sort ‘legitimate’ sources, from those demanding severe punishment against the members of Arran to those relativizing the protest acts. Agustí Colom, for example, pointed out that the actions of Arran ‘do not contribute to the tourism debate’ ( Sust, 2017a), which could be now considered an erroneous statement given the subsequent national/European public debate ensuing from Arran’s ‘radical’ and ‘spectacular’ politics. Economist Miquel Piug (2017), on the contrary, warned against considering these actions as ‘irrational’, arguing that the myth of prosperity surrounding tourism (often associated to economic growth and job creation) needs to be revisited. Mireia Boya, as representative of the CUP party, asked to focus on the violent nature of the ‘economic model’ motivating the protests ^11^, considering the events ‘symbolic acts’ ( Ramos, 2017). Against this type of statement, journalist Alicia Huerta (2017) emphasized the vandalism of the protests, questioning the idea of reading these acts as symbolic but also acknowledging that ‘confetti does not hurt’. Although there are numerous opinions and ‘ideological’ positions about the ‘radical’ acts coordinated by *Arran*, a common statement is that even if these protests are somehow justified, the manner of these acts of dissent was inappropriate and therefore Arran’s actions must be condemned.

***National politics*.** Among the many opinions against Arran, the declarations attributed to Spanish Prime Minister Manuel Rajoy in various media texts are a clear example of the ‘classic’ protest paradigm. Former Prime Minister Rajoy characterized the protesters as ‘extremists’ going against common sense, with clear political agendas (a reference to the Catalan independentism). Prime Minister Rajoy’s statement emphasized the importance of tourism for both GDP (11%) and employment (2.5 million jobs). The ‘where’ and ‘when’ of this declaration seems to be particularly significant. As reported by *El País* ( Vizoso, 2017), this declaration took place during the celebration of the 40 ^th^ anniversary of the hotel chain Hotusa, which reportedly belongs to Rajoy’s childhood friend Amancio López Seijas. Indeed, López Seijas shared his opinion about the ‘anti-tourism’ protests in the same article by *El País*, highlighting both the tolerance and complicity of some local governments (implicitly, this could refer to Mayor Ada Colau) with those who protest against tourism. The businessman additionally pointed out the importance of the labor reform approved by Prime Minister Rajoy, saying that this allowed his company to ‘create positions’ at a ‘reasonable cost’ ^12^.

The Minister of Tourism Álvaro Nadal was likewise quoted in media texts demanding ‘forcefulness’ ( *contundencia*) in both the investigation and sanction of these protest acts. In an interview with *El Periódico de Catalunya* ( Grau, 2017), Nadal explicitly rejects the term ‘tourismphobia’, indicating that he prefers to denote these acts as ‘vandalism’ instead of reading them as ‘protests’. Furthermore, Nadal explains that the issues around tourism are a matter of ‘saturation’ of key services and that regulation and management could help to improve the overall situation of local inhabitants. This statement is incompatible with the position of local groups that actively campaign for tourism degrowth ^13^ and with the requests made by Arran in their online statement (i.e., expropriation of tourist assets, ban of tourist rentals, increased taxes for tourism-related businesses and the improving of working conditions for the workforce in the sector).

***Branding*.** Concerns about the damages to the ‘city-brand’ caused by ‘tourismphobia’ are often mentioned among the many sources in the news articles, varying from local politicians to tourism stakeholders (again the hotel sector or tourism associations such as Exceltur, but also big consortia such as TUI Group). The debate about this branding goes beyond tourism, and various articles have direct references to the candidacy of Barcelona as the host of the European Medicines Agency, which needed to relocate after the Brexit vote (and was later won by Amsterdam, another city experiencing increasing disaffection with global mass tourism). This example shows that branding is not exclusively created for tourism purposes but also for attracting foreign investment, governmental headquarters or high-tech industries. 

In specific, the ‘radical’ protests are perceived as a ‘threat’ to a series of brands, including a) The *Barcelona brand*, as European capital (or *Mallorca brand*, etc.); b) the *Catalunya brand*, as cultural region; c) the *Spain brand*, as ‘sun and beach’ country; and finally, d) the *Europe brand*, as global tourism destination. These brands are intertwined and co-dependent, and while the works of Gaudí might be more associated to the Barcelona brand, other references such as the relative security of the country (i.e. when compared to Turkey and Egypt) are part of the Spain brand. Additional features such as a ‘welcoming nature’ are more ambiguous and seem to apply to the whole set of brands. After the events organized by Arran, the main ‘fear’ was that tourists would read the protests as an ‘attack’ against them and would logically choose another destination, while jeopardizing years of (public) investment in building each specific brand. Maria Salom, delegate of the Spanish Government in the Balearic Islands, was quoted in the *Mail Online* ( Sobot, 2017) saying: “[a] number of irresponsible people cannot damage our image and disturb tourists who visit us.”, a clear statement that decodes protest acts (and especially ‘radical’ protests like those by Arran) as a threat to the carefully advertised ‘image’ of Spain (or Mallorca) and as an inconvenience for paying visitors. 

***Press in the United Kingdom and Germany*.** International media addressed the local discontent regarding the global tourism industry in Barcelona and other Spanish cities, and referring to the term ‘tourismphobia’, there was a sort of *epidemiological narrative* that expressed the fear of the ‘anti-tourism’ protests spreading to other destinations (both at the national level to cities like Madrid and beyond borders to France and Portugal). British press, for example, included a discussion on the value of British tourism for Spain, and while some articles provided information about the ‘reasons’ behind the local discontent (i.e. holiday rentals, overcrowding, ‘unruly’ behavior of Brits abroad), the texts explicitly referred to the economic income generated by British tourists, and at least one article invited Spain to imagine what would happen if British visitors took their money to other destinations ( Gavin, 2017). ‘Tourismphobia’ was also interpreted as a sort of ‘payback’ for the outcome of the Brexit referendum, but this narrative was rare. In German press, there was a relevant discussion about of the results of a poll made to Germans nationals, who expressed embarrassment for the behavior of fellow Germans abroad while conveying understanding of the motives of those who were protesting against mass tourism in Barcelona and other Spanish cities. Considering these local news in United Kingdom and Germany, it could be argued that the ‘anti-tourism’ protests in Barcelona effectively communicated the message in Spain’s two main visitor markets, informing these potential ‘tourists’ about the needs and concerns of local inhabitants and the several negative impacts of their holidays.

***Statements in defense of tourism*.** Most of the arguments in defense of tourism underlined both the economic value in terms of the GDP and its role in job creation. Other ‘positive characteristics’ of tourism, such as the fostering of understanding between different cultures, its contribution to the protection of heritage or its role in education were rarely emphasized in the articles. In short, tourism was generally valued as a global industry, which is a trend that has been previously identified by Higgins-Desbiolles (2006), who argues that this discourse benefits the needs and agendas of tourism stakeholders and local/national governments. Indeed, local and national politicians in the analyzed media texts consistently addressed the need to protect tourism and visitors, although some sources recognized the urgency to find solutions to the many ensuing ‘externalities’ associated to the industry. The solutions included for the Barcelona case, that are similar to those proposed for Venice and Amsterdam, focused on various strategies: targeting ‘luxury’ tourism sectors, tracking tourists with digital technologies, heavy fines for ‘unruly’ behaviors, tourist caps and increased taxes, or the geographical relocation of tourists by creating ‘new tourists attractions’. These solutions are reduced to issues of management and are ‘reformist’ in nature, and in open antagonism with the demands of tourism degrowth made by local groups such as the Association of Neighborhoods for Tourism Degrowth (ABDT) or Arran.

In some articles, journalists included the point of view of tourists concerning their ‘opinions’ or their ‘knowledge’ about ‘anti-tourism’ protests and ‘tourismphobia’. A common argument is that tourists were not aware of any protest against visitors, or they did not understand why locals were unhappy with their presence, arguing that they economically contribute to the city during their stay. As mentioned above apropos the protest paradigm, the quoting of these visitors might work as ‘bystanders’ portrayals that contribute to the delegitimization of the protests, while re-emphasizing the ‘official’ discourse that tourism revenue ‘justifies’ any inconveniences caused by the industry.

A more explicit defense of tourism was observed in petitions endorsed by local stakeholders (i.e., hotel industry stakeholders, tour guides, tourism agencies, etc.), in which these powerful businesses demanded action by the government (both at local and national level) to protect the (perceived) ‘threatened’ industry. Even before the series of ‘attacks’ coordinated by Arran, several members of the hotel sector denounced ‘attacks’ against their establishments (and the total number of events varied according to each media outlet, ranging from two as reported in *El Imparcial* to twelve as reported in *ABC*) ^14^. These powerful business groups advocated for tourism publicly through the media and through lobbying, arguing that the protection of tourism was a sort of ‘common good’ (i.e., ‘we are all tourism’ as expressed by an Exceltur slogan) ^15^.

Finally, an article published by *El Mundo* described the ‘attack’ against the sightseeing bus from the perspective of the Ecuadorian driver, who is quoted saying he only ‘wants to secure an income’ ( *ganarse la vida*). The driver also denies the knife story mentioned by other media, indicating that the protesters used an awl. The article includes a list of other ‘violent’ protests orchestrated by Arran, both in the context of mass tourism and Catalan independentism ( Iglesias & Mucha, 2017). Other workers in the media texts specifically referred to the protest acts by Arran, including the manager of a restaurant in La Barceloneta named Ana Paula Lourenço, who pointed out that “scaring people, making them feel unwelcomed (non grata) is not defending Barcelona, it is damaging Barcelona” ( França, 2017).

***Terrorist attacks*.** The terrorist attacks in Barcelona and Cambrils, which took place on the afternoon of August 17 ^th^, changed the debate about the protest acts. Some articles pointed out the support that both local business and citizens of Barcelona provided to fleeing tourists, characterizing the city as open and welcoming ( Marchena, 2017). Similarly, political acts condemning these terror acts were interpreted as a ‘pause’ in local conflicts, both in regard to ‘tourismphobia’ and the upcoming Catalan Independence referendum. Considering that the attacks happened in one of the most visited spots in the city, the media narratives changed from ‘overcrowding’ of Las Ramblas to the international value of the street ( Cabeza, 2017). The terror act was also perceived as an attack to Western societies, comparing Barcelona to other cities affected by international terrorism ( *yihadismo)*, specifically Paris, London and Berlin ( Ayora, 2017).

A related debate focused on the impact that these terror acts could have in both the branding of Barcelona and the flow of visitors, with sources such as Minister of Tourism Álvaro Nadal pointing out that these events would not damage the reputation of the city as ‘safe destination’ ( Grau, 2017). A similar statement was made by Rafael Gallego from the Spanish Confederation of Travel Agencies (CEAV) in the Spanish website *Expansión*, who pointed out that these acts are a shared ‘risk’ for many European cities, and that these events would not ‘affect the image of Barcelona, Spain or Europe’ ( Arroyo, 2017). Although there are some references to a reported increase in cancellations following the terrorist attacks, experts expressed confidence in the projected growth of tourism numbers, predicting a record year (which it indeed was with circa 82 million tourists).

It is important to highlight that, while international terrorism was considered a sort of ‘single event’ that will not cause significantly impact to the Barcelona brand, the protest acts organized by local citizenship were considered a ‘threat’ to the image and future of the city. This could be read as both delegitimization and demonization of the right to protest, considering that in the long history of dissent against mass tourism in Barcelona, the protest acts have generally been peaceful in nature.

***The 2017 Catalan independence referendum*.** The upcoming referendum on the independence of Catalunya (and the ‘illegality’ of this consultation) was discussed since the early days of the Arran protests, particularly because the group has repeatedly campaigned for the autonomy of the region. After the terror acts that took place in August 2017, the referendum took a more prominent role, allegedly associated with an exodus of several international businesses from Barcelona and the relocation of some cruise ships. With the fear of increased protests of those in favor of independentism, both before and after the referendum, tourism numbers were low and the media narratives discussed this scenario extensively. Although tourism numbers in 2017 were at a record high, several stakeholders of the tourism industry continuously expressed concern about the long-term implications of the political upheaval in the future of the industry, associating the referendum with ‘tourismphobia’.

***The right to protest*.** The main discussion identified in the media texts, both across diverse outlets and throughout the years, was centered on the ‘correct’ ways to protest. The debate questioned the legitimacy of engaging in ‘radical’ politics, which are perceived as ‘vandalism’ and ‘violence’. Although the benefits of mass tourism are frequently mentioned (i.e. employment and economic growth), there is – at various degrees – a recognition that tourism is not harmless, and that it indeed creates a series of grave issues that negatively impact the quality of life of local populations and the environment. The discussion concerning the solutions to these ‘externalities’ tend to focus on technological and management strategies, while discarding other possibilities more in tune with the demands of local activists, such as tourism degrowth.

Although there is not a categorical condemnation of protests or an explicit denial of the right to protest, there is clear evidence of stigmatization, delegitimization and demonization of protesters and their acts of dissent. Various journalists emphasize the circus/carnival atmosphere of the protest acts organized by Arran. The violence of the acts was repeatedly discussed, and this drama does not exclusively refer to ‘the facts’ (which varied from one newspaper to another), but also included *hypothetical situations*. There is a common *‘what if’ narrative* associated to the ‘attack’ against the sightseeing bus in Barcelona, which included statements such as: what if police officers were there? What if there were (violent) hooligans on the bus? What if a tourist defends himself? These potential scenarios contributed to add more drama and spectacle to the ‘news’ article, while further demonizing protesters and their acts of dissent.

As mentioned above, protesters were repeatedly labelled ‘vandals’, ‘aggressive’, ‘angry’ and ‘ignorant’. Several news articles quoted a politician from the People’s Party (PP), Fernando Martínez-Maillo referring to the members of Arran as ‘brainless and insolent kids’ ( *niñatos descerebrados y malcriados*), a clear infantilization of protesters ( Diario Crítico, 2017b). Similarly, a declaration by former Prime Minister Mariano Rajoy referred to protesters as ‘extremists’ ( Vizoso, 2017). While the ‘radical’ politics employed by Arran might justify this depiction for some readers/citizens, the main issue here is that the diversity of social movements and collectives included in the term ‘protesters’ and ‘tourismphobia’ is stigmatized as a whole.

***2018 and beyond ^16^*.** In 2018, Arran continued its activism against mass tourism, and it made the headlines again due to the ‘radical’ politics employed in their protests, with more actions in Palma de Mallorca and Barcelona. In June 2018, the group occupied the famous ‘dragon’ at Park Güell, using chains to tie themselves to the sculpture, as reported by *El País* ( Andrés, 2018), while again using red flares and banners against mass tourism. For this specific protest, their Twitter account included the hashtag #capitalismofòbia (capitalismphobia), which an explicit rejection of the term ‘tourismphobia’. The selection of Park Güell, a space that has been contested between locals and tourists more than a decade ^17^, was evidently a strategy to capture media attention.

Their subsequent acts were still communicated through their Twitter accounts, including a protest near the Olympic village in Barcelona (targeting another sightseeing bus) and another at the airport in Palma (in coordination with the group *Ciutat per a qui l'habita*). In 2019, the group protested again against rental cars in Palma de Mallorca, posting a video on their YouTube channel, this time musicalized with ‘less violent’ music, namely the song “ *In the summertime*” by *Mungo Jerry*. These ‘attacks’ were reported in local and international (British) media and were equally condemned by local authorities and tourism stakeholders.

In 2019, *Arran* Palma tweeted that the members of their organization were facing between 2 and 4 years of prison for their protest at the port of Palma in 2017, for a total of 26 years for the 12 participants. This was perceived as ‘repression’ by the group, and equally condemned by other left-wing organizations such as *Alerta Solidària*.

In 2020, after the coronavirus pandemic forced the cancellation of the majority of flights and cruises, and left Barcelona and Palma de Mallorca reasonably emptied of tourists, Arran continued its online activism publishing a statement on their own website, highlighting once again the dangers of the dependency on the global mass tourism industry, which in the context of the pandemic meant added health risks, precarious work and lack of access to affordable housing.

### An interview with a spokesperson from Arran

Before concluding this paper, this section offers some additional information resulting from a short interview that took place with a spokesperson from Arran in Barcelona in early January 2020 ^18^. The interview was semi-structured, and the main goal was to contrast the information published by both local and international media. When asked about the “three main negative side effects” associated to mass tourism in Barcelona, the spokesperson from Arran identified ‘four’: a) gentrification and lack of access to affordable housing, which causes the expulsion of local inhabitants; b) the environmental impact of tourism, particularly in relation to cruise tourism; c) overcrowding and appropriation of public spaces, creating a sort of ‘theme parks’; and finally d) the economic model created by the industry, based on precarious work and exploitation. The answers were similar to the ones given by other ‘less radical’ activists, and instead of fitting the media characterization of the members of Arran as ‘childish’ or ‘ignorant,’ ^19^ the spokesperson from Arran offered a ‘fact-based’ explanation (and one which could be supported by former studies on the impacts of mass tourism in Barcelona). For example, the environmental impact of cruise tourism in Barcelona has been documented and reported by *Transport and Environment* , in which the city occupies the first position in cruise-related air pollution (followed by Palma de Mallorca and Venice). Another important detail about this answer is that it was consistent with the various statements made by Arran in their protest acts over the past years, emphasizing the link between global mass tourism and precarious work or the shortage of affordable (social) housing. 

In the interview, Arran identified itself as an Independentist and left-wing organization, pointing out that their activism was not mainly oriented towards ‘mass tourism’, but that the industry was “one of the most evident contradictions” of the current capitalist system. When asked about the role that media plays in portraying the protest acts against mass tourism (in general and about Arran in specific), the spokesperson said that media (although not all) generally takes the side of the tourism industry:

*Arran Spokesperson:* It is also true that not all the media play this role… In the end, it depends on who they serve… But it is true that a big part of the media, in the end, they side with the status quo and the system… and therefore, any protest act that goes beyond a certain margin of… the social consensus… and that is a direct affront against the economic interests… Well, it exposes itself to this type of criminalization… by the media…
(Translation by the author)

Arran valued the use of digital channels and social media and considered it “an opportunity for the organization to decide which message communicates or not”, although these spaces are “not neutral”. This is relevant for the purposes of this paper because Arran was able to insert themselves in the mainstream media after posting their videos on their social media accounts, and they had the advantage to relatively shape the timing of the ‘news’ (while creating expectation). Moreover, Arran also opposed the ‘solutions’ proposed by local authorities and tourism stakeholders that were oriented towards reforming the current tourism management (i.e. those aiming at creating new tourist venues or attracting luxury tourism, etc.). On the contrary, the group insisted in the need to protect vulnerable groups and to campaign for tourism degrowth (“a change in the economic model”).

When asked about the violence used by the group in their protest acts, the spokesperson from Arran called into question the definition of ‘violence’, pointing out the political bias inherent in the concept:

*Interviewer: Do you consider that the type of protest that you do is violent?*
*Arran Spokesperson:* [SIGHS] In a way, I would say that… And considering the examples that we are talking about [THE ACTION INVOLVING THE SIGHTSEEING BUS AND THE LATER PROTESTS AT PARK GÜELL], I would not say violence, I think it is used in a biased manner. But it is also true that we want to antagonize this idea… that violence delegitimizes, but that the violence of the state, the violence of the system, is both tolerated and accepted. In the end, we are talking about who has the legitimate use, or legal [USE], of violence and… and… in the end… Violence could be a tool for the working and oppressed classes, against the system. And we don’t strictly condemn, in this sense, any action for the mere fact that it implies violence. But it is true that… when we talk about chaining ourselves to the dragon at Park Güell, clearly, we could not speak of violence… And, at the most, we are talking about actions that break the social consensus, and the social peace, and all that… It is easily labeled as violence. But we are talking about objects, so in that sense, no… (Translation by the author)

To conclude, Arran indicated that this ‘violence’ and ‘spectacular’ forms of protest was needed to capture media attention and promote social debate, although these protest tactics do not produce immediate or significant changes:

*Arran Spokesperson:* It is evident… I mean, in the end… The motive that it pursues… It is clear that sometimes this type of actions does not create a material change, at that precise moment… But what we have analyzed is that it allows us to put some topics on the table… and in the public agenda… that we could not have done in another way… I mean, if we stand one day with a banner in front of some place, maybe this does not reach the media… But the action of the sightseeing bus does, and it generates interest, and it comes out... It allows us to debate and talk about these issues. In this sense, yes, it manages to capture the attention. (Translation by the author)

This interview provided a needed insight into the ‘rationality’ of the young collective Arran *,* which is relevant for various reasons. First, the members of the collective – or at least their spokesperson – seem to be strategic in approach towards their acts of dissent, knowing what kind of tactics will work to attract the attention of both media and local or national authorities, which relates to the theory of both political and media opportunities. Following Meyer (2004: 128), by paying attention to the political context and the rules of the game (the *structure*), the members or Arran were able to decide their ‘violent’ and ‘creative’ tactics (the *agency*). Second, the protest acts were a *series of events* happening both within a city (in various neighborhoods in Barcelona) and in other places suffering similar side effects of global mass tourism (i.e. Mallorca, Bilbao, Valencia, etc.). As Cable (2016) has identified for groups protesting airport expansions and climate change, this strategy allows the protester to augment their media opportunities, which might help to keep political opportunities open.

From a spatial analysis ( Parkinson, 2012), the protest acts by *Arran* challenged these *sites of consumption* ( Routledge, 2017) that were also *sites of assumption*. They interrupted the logic of these spaces and used them for their dissent, aiming at re-signifying them as *sites of destruction* (of the local way of life, social ties through expulsion, etc.). A restaurant or a sightseeing bus might seem ‘neutral’ at first, but they ‘represent’ deeper societal changes that privilege a group of users (tourists) over other (local inhabitants). Although this spatial analysis is not part of the goal of this paper, it seems that Arran has planned each of their protest acts carefully, balancing costs and benefits and choosing sites that would help them to promote their cause (particularly evident in their protest act at Park Güell in 2018).

In relation to the alleged ‘violence’, ‘vandalism’ and even ‘trespassing’ of their protest acts, the notion of civil or democratic disobedience could be useful to analyze Arran’s ‘radical’ politics. There is a long debate about civil and democratic disobedience and their limits ^20^ ( Alakbarova, 2019; Celikates, 2016; Lefkowitz, 2007; Markovits, 2005), there are scholars who argue that these political actions should be ‘public’ and those who challenge this requirement, there are those who emphasize that protesters should be willing to accept punishment (for breaking the law) while others strongly oppose the idea of protesters being punished for trying to fix structural deficits within the democratic system. There is a range of interpretations on the legal use of ‘violence’ and on the definition of ‘radical’ politics. To some extent, the protest acts orchestrated by Arran since 2017 could be read as a form of civil or democratic disobedience, and as way to expand democracy and to (forcibly) include ‘disenfranchised’ voices in both opinion- and will-formation and decision-making processes.

## Conclusions

This qualitative media analysis illustrates the complexity of the public debate about global mass tourism and its ‘externalities’ in Barcelona and other Spanish cities. In terms of local politics and management of tourism, the acts of dissent organized by Arran fueled an extensive debate that crossed borders and engaged a diversity of sources, including politicians (local and national level), representatives of the hotel sector, tourists, scholars, stakeholders of the tourism industry in Europe, and local activists and inhabitants of Barcelona/Spain. There are three main conclusions regarding the media narratives discussed in this paper:

- **The relevance of national and local politics:** The management of tourism and the understanding of the acts of dissent organized by Arran could not be separated from the wider political discussion on the Catalan Independence; or from the power struggles between local/national parties in Barcelona (or related Spanish cities) and their own political and economic agendas. In this sense, as Hornig (2017) has suggested for the case of airport protests, there seem to be a disparity between the scales in which both the benefits and the externalities of mass tourism are distributed, which are a form of *vertical asymmetric policies*: National and regional stakeholders might prioritize the economic value of the industry because they are less impacted by the negative effects, which remain a local issue. The role of lobby groups (particularly evident in the case of the hotel industry) is also significant, given that these groups have direct or privileged access to both opinion-formation and decision-making processes through both their depiction in the media and their proximity to political power.

- **A ‘soft’ use of the protest paradigm and of marginalization devices:** Considering the vast diversity of media outlets included in this study, it is not possible to identify a unified discourse about the protest acts. However, there is clear evidence that protests are stigmatized or deemed ‘inappropriate’. The term ‘tourismphobia’ homogenizes the diversity of protest strategies and social movements, emphasizing the idea that this is an ‘emotional response’ to changing circumstances in a specific context (i.e. the neighborhood, the city, etc.). Social movements have campaigned against this buzzword, which was repeatedly employed by journalists, politicians and tourism stakeholders ^21^. Undeniably, social movements might be motivated by emotional factors (for example, the feeling of loss of their daily spaces), but this does not imply that these collectives do not engage in serious analysis and research regarding the side-effects of mass tourism (including varied activities such as documentation of the impacts of tourism or specialization in local, national and European policies). Protesters are oftentimes labeled as ‘radicals’, ‘extremists’ or ‘absentminded’ but the main narrative seems to be about ‘the correct ways to protest’. ‘Radical’ politics such as those used by Arran are considered ‘illegitimate’ strategies, forms of ‘vandalism’ and ‘violence’ (or even ‘terrorism’ ^22^). These strategies are in sharp contrast with those employed by powerful lobbies (for example, the hotel sector), which consist of press releases or written petitions (and one could speculate that this also includes a fair amount of lobbying).

- **Oversimplified conflicts and incomplete/inaccurate information:** There seems to be a ‘pattern’ in many news articles, which consist of: a) a general statement about the value of tourism, b) description and information about the protests, c) sources both in favor or against the dissent acts, and d) potential solutions to deal with ‘overtourism’ and improve the management of the industry. Few articles provided an extensive and highly detailed description of the issues behind the protests, while generalizations were more common. This could be the outcome of the central role played by news agencies or the lack of resources to produce high-quality ‘news’. Incomplete or inaccurate information was also found, including references to events that have been cancelled, or repetitive information that has been questioned by other sources. A good example of journalism has been identified in the *Diario de Mallorca*, which dedicated a couple of articles to describe and dispute the false information published by other local and international media in relation to the protest acts by Arran. Both oversimplified conflicts and incomplete/inaccurate information might depend on the dynamic of news production, which are affected by constraints of time, personnel, budget or availability of sources.

In short, the quality of the ‘news’ could be significantly improved by including the voices of local activists and by dedicating more resources to explain the complexity of the global mass tourism industry (which only a few long and detailed news articles do). In the case of ‘radical’ politics, journalists and editors are advised to question statements from official sources that openly stigmatize protesters, or those that further perpetuate the idea of protests as ‘violent’ and ‘inappropriate’ instead of recognizing them as a fundamental democratic right.

## Data availability

Zenodo: RIGHTS UP - Table with references for media texts regarding protests against mass tourism in Venice, Amsterdam and Barcelona (2014–2017). http://doi.org/10.5281/zenodo.4740404 ( Araya López, 2021a)

This project contains the following underlying data:

- RIGHTS UP - Codes for Qualitative Analysis.pdf

- RIGHTS UP - Media Texts (01.01.2014 - 31.12.2017).csv

Zenodo: RIGHTS UP - Interviews with activists protesting mass tourism in Barcelona [Transcripts - English translations]. https://doi.org/10.5281/zenodo.4739716 ( Araya López, 2021b).

This project contains the following underlying data:

- RIGHTS UP - BAR01 - Defensem Park Güell.pdf
- RIGHTS UP - BAR02 - Las Kellys.pdf
- RIGHTS UP - BAR03 - Arran.pdf
- RIGHTS UP - BAR04 - Barris Per Viure.pdf

Data are available under the terms of the Creative Commons Attribution 4.0 International license (CC-BY 4.0).

## Footnotes

^1^ Hughes (2018) offers a detailed analysis of the role played by the CUP party in the protests against ‘mass tourism’ in Barcelona, linking it with broader political processes such as the conflict between Catalunya and the Spanish state over the Catalan Independence. This historical background is also relevant for this paper and will be discussed in this article.

^2^ The validity of a media text was defined accordingly to its thematic relation to the issue of tourism and dissent. Some excluded articles included biographies, travel guides, news on literature and art, and other news that included the ‘keywords’ but were not related to the issue of mass tourism. The period of study was selected following the academic literature on dissent against mass tourism apropos the three cities of the study (i.e. Venice, Amsterdam and Barcelona), complementing this media analysis (2014–2017) with participant-observations, fieldworks and in-depth interviews (during the years 2018–2020). A post-pandemic analysis of both media texts and the new strategies for dissent adopted by these social movements is recommended for future studies. Some alternatives to the database Factiva include Proquest and LexisNexis.

^3^ A table detailing the selected media texts, including the codes used for the qualitative analysis, is available in the open repository Zenodo: **10.5281/zenodo.4740404**

^4^ This includes articles in Catalan that are a literal translation of the same article published in Spanish or English, articles written by the same author but published in different newspapers with minimal variations, or articles identified by the Factiva algorithm as “duplicates”.

^5^ A non-literal English translation of the transcripts of these four in-depth interviews is available in the open repository Zenodo: **10.5281/zenodo.4739716.**Any personal and sensible information that could damage the human participants or that could indirectly contribute to their identification has been redacted.

^6^ This moratorium was approved by a coalition between Barcelona en Comú and the ERC (Republican Left of Catalonia) party, while the CUP party abstained in protest, as reported by *El País* ( Blanchar, 2017). The CUP campaigned for tourism degrowth across Barcelona, which is an important statement because according to several social movements, including activists from the nearby municipality of L’Hospitalet de Llobregat (personal interview, January 2020), the ‘hotel ban’ in Barcelona only managed to geographically relocate the problem to the ‘periphery’. These activists denounced that the identity of L’Hospitalet was being lost, with the community becoming only an appendix of Barcelona (due to its integrated transport system and geographical proximity). This process could be defined as *‘overtourism by proxy’* and it is also identifiable in the hotel ‘boom’ in Mestre, given its proximity to Venice (although in this case Mestre and Venice constitute the same political unit, while L’Hospitalet de Llobregat has its own local government).

^7^ In an interview with a spokesperson from *Arran* that took place in Barcelona in January 2020, the use of a knife during this protest was also denied. This interview is discussed in the last section of this paper.

^8^ Several attempts were made to interview this witness to verify this specific statement about ‘terrorism’, but the source was not able to collaborate.

^9^ A theoretical and critical examination of the so-labeled ‘anti-social’ behavior of tourists in Venice, Amsterdam and Barcelona has been published elsewhere ( Araya López, 2020), focusing on the strategies adopted to promote ‘respect’ among visitors and the punishments used to prevent such transgressions.

^10^ Many local activists consider these terraces a form of ‘privatization’ of public spaces. Mansilla (2018) offers an analysis of the terraces in pre-pandemic Barcelona, with a recent update on the subject in the aftermath of the pandemic ( Mansilla, 2020).

^11^ A similar statement is given by the Arran spokesperson in the interview included in this paper.

^12^ Local activist groups such as Las Kellys in Barcelona, a collective of (female) hotel housekeepers who fight against precarious work linked to outsourcing businesses that provide cleaning services to hotels and tourist rentals in Spain ^12^, have frequently denounced the many abuses in the hotel sector, including explicit references to the impact of Rajoy’s labor reform in their personal lives (personal interview in January 2020). Similarly, Hughes (2018: 474) refers to the position of the CUP party in relation to jobs and tourism, pointing out that work in the sector is ‘seasonal, precarious and part-time’, including payments ‘in cash’ and other illegal or questionable practices. The full transcript with the activist from Las Kellys in Barcelona also confirms these precarious work conditions. It is important to emphasize that the statements of the collective Arran have been consistent in their commitment with the ‘working class’, and that this is a central theme in their protest acts and their political manifestos.

^13^ Hughes & Mansilla (2021) have recently published a political discourse analysis on the relationship between dominant tourism narratives in Spain (mostly held and promoted by local/national authorities and tourism stakeholders) and the degrowth campaign promoted by the collective *Association of Neighborhoods for Sustainable Tourism (ABTS),* which is now known as *Association of Neighborhoods for Tourism Degrowth (ABDT).*

^14^ In the interview with a spokesperson from Arran, the group denied their participation in these incidents.

^15^ This idea supports the analysis of the ‘discourse of conviviality’ proposed by Hughes & Mansilla (2021), which states that local Spanish people are expected to create a welcoming environment for tourists (instead of meeting them with hostility).

^16^ These events are included here as additional contextual and historical background. As mentioned above, the analysis of media texts was delimited to the period from January 2014 to December 2017.

^17^ An interview with an activist from the collective *Defensem el Park Güell* took place in January 2020. See also: Arias-Sans & Russo (2017).

^18^ A total of 24 activists in the three cities of this study (i.e. Barcelona, Venice and Amsterdam) were interviewed to discuss their activism, the current impacts of tourism in the city, their emotions, the role of the state and the local government in solving the ‘tourism crisis’, etc. Other interviews planned, including some with tourism stakeholders and governmental authorities, were cancelled due to the coronavirus pandemic.

^19^ A few news articles included a personal ‘critique’ against a member of Arran named Mar Ampurdanès, describing her ‘wealthy’ social background (a house with a swimming pool). This ‘profiling’ of activists and the inclusion of details of their private lives has also been identified by Cable (2016) for environmentalist groups campaigning against air transport (airport expansions) in United Kingdom.

^20^ The discussion on civil and democratic disobedience is long and includes references to legal scholars and theorists of social justice such as John Rawls and Joseph Raz, political figures and supporters of ‘peaceful’ direct action such as Martin Luther King, Nelson Mandela or Mahatma Gandhi. The scholars included in the references offer a contemporary and detailed analysis of this academic, legal and political debate.

^21^ In the middle of this long public debate on ‘tourismphobia’, several local and national authorities campaigned against the use of this term because it could have the side effect of keeping tourists away.

^22^ Hughes & Mansilla (2021) have also referred to phrases used by local resistance in Barcelona, including “hipsters and tourism, new forms of terrorism”, which could suggest that also the label of “terrorism” is politically contested, with both pro-tourism and anti-tourism movements trying to symbolically discredit the other.
