# Supplementary material for: A summer of phobias: media discourses on ‘radical’ acts of dissent against ‘mass tourism’ in Barcelona
Source: Open Res Eur. 2021 Jun 10;1:66. [Version 1] doi: 10.12688/openreseurope.13253.1 (PMC10445860; doi:10.12688/openreseurope.13253.1)
Supplement: Peer review history

# Peer review: Reviewer response for version 1

**DOI:** 10.21956/openreseurope.14323.r27160

**Published:** 2021-08-02

**Authors:** Alice MATTONI

**Affiliations:** 1 Department of Political and Social Sciences, University of Bologna, Bologna, Italy

Review Open Research Europe – A summer of phobias

 The paper "A Summer of Phobias" by Alexander Araya Lopez investigates the news media representation of protests against 'mass tourism' that the grassroots activist group Arran organised in Barcelona and other cities across Spain in the summer of 2017. Overall, although not stated explicitly, the research question to which the paper seems to answer (cfr. page 3 of the paper) is:

 RQ: how 'radical' protest acts are represented in (mainstream) media discourses?

 The paper employs discourse analysis on a corpus of both national and international news reports about the protests to answer such a research question. It illustrates the presence of different discourses within the news media representation of protest, of which the most relevant are the following. First, the connection between Arran's protest and the local and national political debate. Then the stigmatisation of protests, with Arran represented as an illegitimate political actor. Finally, the oversimplification of the representation of the conflict around 'mass tourism', also due to the use of incomplete or inaccurate information.

 I believe the paper offers some valuable insights on our understanding of news media representation of protest in Spain and, to a more limited extent, at the international level of the German and British press.

 However, I have some suggestions to improve the author's work on this subject matter. Please note that all my comments are constructive and sympathetic towards the author's research endeavour.

 My first two comments are strictly related to the knowledge I have of social movement literature, primarily when it deals with the relationship between social movements, protest and media at large. I make these two comments because, through its leading research question, the paper seems to position itself somehow naturally in the realm of social movement studies rather than on media studies, generally speaking. It is for this reason that I believe these two comments might be helpful to the author.

 The first is a minor comment related to using the 'social movement' concept in the paper. Social movements are processes that are sustained and organised by movement organisations and include, among other forms of collective actions, various forms of protest (cfr. della Porta and Diani 2020). However, when reading the paper, I felt that the author employs social movements as synonymous with movement organisation and mobilisation in other parts of the paper. Therefore, for clarity, I would revise the paper keeping in mind the distinction between social movements, which is an overall social process, the collective actors that participate in them, which are movement organisations, and their actions, which are protests. 

 The second comment instead concerns the overall literature on how news media represent protest. There is rich literature in this regard, and I believe the paper might position itself within such literature, giving a substantial contribution to it. However, the author does not review this literature and does not connect to it and its main findings. These studies focus on selection bias (i.e. McCarthy *et al *1996, McCarthy *et al *2008, Oliver and Maney 2000) and on representation bias (i.e Smith *et al *2001) related to protest. Also, there is rich literature on the so-called protest paradigm that he also employs in his paper and the related news media analysis (cfr. Gil-Lopez 2020, also for her review of the protest paradigm literature).

 In this regard, I did not clearly understand the relationship between the section *'What is 'the news'? Newsworthiness and the Consumption of Media' *and the overall scope of the paper and its leading research question. The section is well written and includes various valuable insights, also on the blurring lines between news producers and news consumers. However, I could not understand how such a section helps the author in paving the ground for his subsequent discussion of methods and findings. It seems that a literature review on biased news media coverage of protest would be more appropriate to ground the author's empirical work and the related results.

 As for the corpus of data used for this paper, the author should clarify a few aspects. First, the overall corpus of data gathered in the research project RIGHTS UP range from 2014 to 2017 and includes 723 valid articles. Second, however, the paper deals with protests organised by one movement organisation – Arran – during the summer of 2017. Therefore, I think it would be good to know: 1) how many articles have been analysed with this regard and 2) from which sources (local, national and international; in the case of the former, from which region in Spain; in the case of the latter, from which country). I think a table would be sufficient to illustrate this point.

 Then, I have a more general comment on methods. The author mentions the protest paradigm and the marginalisation devices, which I think would be a relevant heuristic for analysing the corpus of data. However, the author prefers to employ discourse analysis to find out which are the most relevant discourses on the protests in the summer of 2017. Then, he also adds that the protest paradigm and several marginalisation devices were found in the corpus of data, although in a 'softer' version (cfr. page 5 of the article). It is not clear to me how the author reached this conclusion since he did not employ the protest paradigm dimensions and the marginalisation devices as the main elements of his coding scheme, which was rather developed deductively from the literature.

 I have another comment related to the data analysis that I think deserves some clarification: the author states that he engaged in discourse analysis. However, then it is not clear how the author operationalised discourse: which dimensions of discourse he took into consideration precisely? I have to be honest here, of course: I do not know and could not retrieve the chapter by Tonkiss (2004) that the author mentions in the paper. However, considering the three aspects that the author mentions (cfr. a,b and c page 5, with particular reference to the first one), could we instead see the analysis as more related to thematic analysis (Braun and Clarke 2012) than to discourse analysis strictly speaking? If not, I believe it would be essential to explain why and to discuss more in-depth in which sense this analysis is related to the tradition of discourse analysis. Also, it is unclear how the author paid attention to silences in his analysis: were they somehow coded for each document/theme (it does not seem so from table 1)? If not, how did the author analyse silences and how he included them in discussing the results?

 The findings then present the protest events that happened in the summer of 2017. The discussion in this section is particularly thick, with rich details about the various discourses and the representations of the actors. However, it discusses neither the protest paradigm nor the marginalisation devices in detail, in any case. For this reason, I already stated above that there seems to be a disconnection between the theoretical framework of the article and the actual analysis that the author conducted.

 About the section that presents the various discourses, I would leave out the sub-section about how the press represented the local protest in the UK and Germany. This sub-section, indeed, does not seem to cover a theme per se, but rather how other news media than the Spanish covered the local protests: I imagine that some themes were more prominent than others in the international press coverage. It might be good to have a whole section on this so that the national and international press coverage of the protests might be compared as well about its prevailing themes. The finding here would be related to assessing (or not) if the same protest paradigm/marginalisation devices were at work in the national and international protest coverage. Overall, I think this is a missed opportunity of the paper.

 I would also leave out from this general section related to the discourses that emerged from the analysis the last sub-section, entitled "2018 and beyond", that might be better positioned in the paper's conclusions to explain how protests developed after the time under investigation.

 Finally, the author includes a section that presents an in-depth interview with one of Arran's spokesperson. The scope of this section, if I understood well, is to contrast how national and international media represented Arran's protests. Overall, this is fine, but I do not see how this is consistent with the research question that the author wanted to address in the paper. The latter is indeed strictly related to how news media represent protests and, more specifically, the main discourses that they produce around them and the contentious issue they tackle. I believe the in-depth interview might be the basis for a different paper related to Arran's communication strategies and their perception of mainstream media coverage about their protests. Of course, in this case, it would be better if the in-depth interview would be included in a broader corpus of interviews with other Arran's members and triangulated with Arran's materials and accounts of the protest to be retrieved via social media platforms.

 If the author wants to keep this section in the paper, though, he should explain why it is there, as the reason currently given in the paper is not convincing and is not consistent with the main research question.

 Concerning interviews, it is unclear why the author refers to four interviews with key informants in the section related to ethics. Then in the paper, only one interview seems to be presented and discussed.

 To conclude, I believe that the paper is promising, addresses a relevant issue – both theoretically and empirically – and rests on a solid corpus of data. Beyond some minor comments, my main suggestions concern three significant points that I hope the author might address to strengthen the paper further:

1. The positioning of the paper in the overall literature on mainstream media and social movements, with particular reference to those scholars who explored how mainstream media represent protest.
2. The consistency between the research question, the analytical framework and the method of analysis that the author applied.
3. Some other remarks on the structure of the paper and the missed opportunity to compare national and international coverage of the local protests.

If applicable, is the statistical analysis and its interpretation appropriate?

Yes

Is the study design appropriate and is the work technically sound?

Partly

Is the work clearly and accurately presented and does it engage with the current literature?

Partly

Are the conclusions drawn adequately supported by the results?

Partly

Are sufficient details of methods and analysis provided to allow replication by others?

Partly

Are all the source data and materials underlying the results available?

Yes

Reviewer Expertise:

Social movement studies, with specific expertise on the relationship between media and social movements, movement organizations and protest.

I confirm that I have read this submission and believe that I have an appropriate level of expertise to confirm that it is of an acceptable scientific standard, however I have significant reservations, as outlined above.

## References

[1] : Images of Protest: Dimensions of Selection Bias in Media Coverage of Washington Demonstrations, 1982 and 1991. *American Sociological Review* .1996;61(3) : 10.2307/2096360 10.2307/2096360

[2] : Assessing Stability in the Patterns of Selection Bias in Newspaper Coverage of Protest During the Transition from Communism in Belarus*. *Mobilization: An International Quarterly* .2008;13(2) : 10.17813/maiq.13.2.u45461350302663v 127-146 10.17813/maiq.13.2.u45461350302663v

[3] : From Protest to Agenda Building: Description Bias in Media Coverage of Protest Events in Washington, D.C. *Social Forces* .2001;79(4) : 10.1353/sof.2001.0053 1397-1423 10.1353/sof.2001.0053

[4] : Political Processes and Local Newspaper Coverage of Protest Events: From Selection Bias to Triadic Interactions. *American Journal of Sociology* .2000;106(2) : 10.1086/316964 463-505 10.1086/316964

[5] : Mainstream Protest Reporting in the Contemporary Media Environment: Exploring (In)Stability and Adherence to Protest Paradigm From 1998 to 2017. *Journalism & Mass Communication Quarterly* .2020; 10.1177/1077699020984783 10.1177/1077699020984783

[6] : Thematic analysis. 2012; 10.1037/13620-004 57-71 10.1037/13620-004

# Peer review: Reviewer response for version 1

**DOI:** 10.21956/openreseurope.14323.r27083

**Published:** 2021-07-12

**Authors:** Neil Hughes

**Affiliations:** 1 Department of Modern Languages and Cultures, School of Cultures, Languages and Area Studies, University of Nottingham, Nottingham, UK

I welcome this opportunity to review this excellent paper. In it, I adopt the stance of critical friend and taking my lead from Araya López’ article, ‘prosumer’, which is a subject position he mentions at the beginning of his paper. In the position of critical friend, I highlight the reasons, of which there are many, that readers with interests in a range of subject disciplines might find the paper compelling reading. From this position, I also outline some criticisms relating firstly to the author’s treatment of the Catalan political movement, Arran, a collective with links to the left-wing nationalist Candidatura d’Unitat Popular, CUP (Popular Unity Candidacy) and secondly, to his use of evidence. In terms of the prosumer role, I draw on research of my own to add weight to some of the author’s main claims and secondly, to substantiate some of my own arguments. 

 On the positive side, this is a highly readable, and insightful paper that articulates well with the growing body of literature looking at aspects of tourism from a critical perspective. While it clearly situates the study within the Media Studies field, there is plenty here to engage those with interests in other subject areas including Social Movement Studies, Tourism Studies, Spanish Political Studies, and Critical Discourse Analysis (CDA) to name just a few. In terms of the concepts used to explore the different media accounts of the wave of anti-tourism protest that has swept across both Barcelona and Catalonia more widely since 2014, these are clearly set out by Araya López. They are identified as the ‘protest paradigm’ and ‘marginalisation devices’, both of which are clearly explained and deployed effectively in the article.  

 Regarding methodology, the methods used to identify a corpus of national and international media texts addressing different aspects of the protest cycle as well as the process used to analyse them are explained in enough depth for students or academics with interests in Media Studies to be able to replicate them in their own studies. Araya López uses the corpus effectively to map the protest events, to explore their links to phenomenon, such as Airbnb, to analyse the regulatory response to local discontent, and to draw parallels between the anti-tourism activism witnessed in Catalonia and activism witnessed in other parts of Spain including in the Basque Country. The mapping process reveals a protest cycle against over-tourism that began to rise in 2014 and reached its apogee in 2017. As well as exploring the wider context within which it was produced, the analysis of the media discourse analysis reveals the dominant narratives produced based on the provocative neologism, ‘tourismphobia’ and the somewhat more neutral concept of ‘over-tourism.’ For anyone interested CDA, it also explains the role played in the media representations by linguistic devices such as ‘what if’ rhetoric, which the author argues is used to demonise the protestors.   

 One of the author’s key insights concerns the political use made of the term ‘tourismphobia’ by journalists, national politicians, and important stakeholders in the tourism industry. Araya López correctly asserts that the arbitrary application of the term ‘tourismphobia’ to all acts of anti-tourism industry protest has meant firstly, that the ‘complexity and breadth of the coalition of forces that has united against over-tourism has been lost’ and secondly, that all protest, even entirely lawful and peaceful political activity, has been ‘stigmatised’. The discussion of elite actors’ use of tourismophobia to describe all acts of protest against tourism’s excesses provides a bridge to the wider literature discussing these issues. According to Milano et al (2019), for example, the use of ‘tourismophobia’ is part of a concerted effort by the media and other elite actors to divert attention away from legitimate social movement concerns such as tourism workers’ pay and conditions:  

*"the social media bubble on tourismophobia that exploded during the summer of 2017 coincided  with the denouncements for improved labour rights in the hotel sector by housekeepers’ unions  and professional associations" (2019: 11).*

 In terms of the complexity and breadth of the anti-tourism industry movement, as Hughes (2018) pointed out, popular dissatisfaction has fueled calls from an increasingly vociferous coalition of grassroots groups, in particular, in Barcelona for city authorities to ensure that neighbourhoods remain accessible and liveable for local people. As Hughes and Mansilla (2021) argue in a more recent contribution, there have also been attempts to unite the disparate anti-tourism struggles that have broken out in neighbourhoods across Barcelona into a common front against the economic, social, and environmental costs of a process of city redevelopment that has transformed Barcelona from an aging industrial port city into a 'not to be missed' destination for many discerning travellers. This led, in 2017, to the setting up of the Assemblea de Barris per un Tursime Sostenible or Neighbourhood (Assembly for Sustainable Tourism) that organises coordinated action against the negative impact of mass tourism across Barcelona. As Hughes and Mansilla explain (2021), it integrates more than 30 neighbourhood groups, assemblies, and movements that are united by their rejection of what they see as the neoliberal ‘touristification’ of their neighbourhoods and commitment to practices and values such as assemblyism and consensus-based decision-making, environmental responsibility, degrowth, and the ‘right to the city'. Many of those involved in anti-tourism contestation are also political party activists, most commonly in either Barcelona En Comú (Barcelona in Common), a citizens’ platform that currently governs the Barcelona City Council, or the more explicitly anti-capitalist Candidatura d’Unitat Popular, CUP (Popular Unity Candidacy).  

 The emphasis in ABTS’s discourse on the link between neoliberal restructuring, over-tourism, and the need for degrowth are articulated by ABTS activists in their contribution to the academic literature that has emerged looking at these issues in recent years. In his collaboration with the academic, Agustín Cocola-Grant, for example, the ABST activist, Daniel Pardo (2017), identifies neoliberal measures introduced both prior to and, in particular, in the wake of the 2008-2013 financial crisis as one of the main reasons for the spectacular increase in tourism stays in Barcelona, which they estimate at more than 30 million a year. Such measures include the relaxation of urban planning regulations limiting hotel and holiday accommodation in the historical city centre; measures to incentivise tourism businesses such as tax breaks and the loosening of licensing restrictions on businesses; air transport deregulation; labour market reforms, and a lax regulatory environment for tourism accommodation platforms, such as Airbnb, all of which, they argue, have contributed significantly to Barcelona’s ‘touristification’.   

 As regards the accusations of violence by the Spanish and international media, since the beginning of the cycle, the protests organised by movement actors have been almost entirely peaceful. In Ciutat Vella, for example, the urban collective Fem Plaça (Let’s Make the Square) has developed a multi-faceted, non-violent campaign based on condemnation of the impact of over tourism on the neighbourhood’s squares and plazas. As an alternative, The Fem Plaça Collective calls for greater regulation by the city and regional authorities and an approach based on communitarian principles that reclaims privately appropriated public space. This view is reflected in the slogans that adorn The Fem Plaça Collective’s posters and banners in the streets and squares of Ciutat Vella. These make wide use of the Catalan imperative form to call for citizen-generated urban interventions including the taking over and repurposing of public space lost to commercial activity as in the case of, ‘Recuper l’espai public com a lloc de convivencia (Take back public space as a place of co-existence) (Bruttomesso, 2018). The combination of imperatives and the use of first-person plural forms apparent in the collective’s name and in slogans such as, ‘Fem Plaça, Fem Platja’ (Let’s Make the Square, Let’s Make the Beach), challenge the commodification of space and help build solidarity within Ciutat Vella and with other neighbourhoods involved in tourism contestation. Such language use contributes to the construction of wider social movement identity and helps build cohesion across the diverse network of actors involved in the protests (Hughes, in press). 

 The one exception to the peaceful trend seen in most of the anti-tourism industry activism witnessed in Barcelona and across Catalonia, has been the acts of contestation organised by Arran. In them, Arran has resorted readily to acts of violence and vandalism against tourists and tourism interests. These have often been accompanied by the deployment of unedifying slogans such as ‘tourist you’re the terrorist’ and ‘leave or die’ that use ‘us and them’ rhetoric, ‘other’ tourists as an existential threat, and warn of the consequences to tourists if they remain in the city. Such slogans have functioned as a ‘call to arms’ to young activists to take action against tourists to protect the city and return it to its people. As Araya López discusses, in August 2017, for example, a group of young activists were reported to have stopped a sightseeing bus close to the Camp Nou football stadium, slashed its tyres and painted the slogan, “tourism kills neighbourhoods” on its side. Videos of the attack were uploaded to YouTube shortly after the events accompanied by the caption, “mass tourism kills neighbourhoods, destroys the territory and condemns the working class to misery”. In other incidents, for-hire bicycles had their tyres punctured and several hotels and restaurants were paint-bombed. Again, the video’s accompanying slogans appear to legitimise the vandalism as do subsequent justifications for the attacks issued by the activists in which they describe their actions as acts of “self-defence” against the tourism invasion. 

 Given the tactics used by Arran activists, I find it surprising that a spokesperson from this organisation was chosen by the author to contest the claims of ‘tourismphobia’ and unfair treatment by the media. Despite their protestations, it is clear from their attempts to defend their actions as a legitimate means to attract media attention, that Arran has very few moral qualms when it comes to tactics that ‘other’ tourists, place people’s lives in danger and destroy property. It is also important to point out that it is not just the mainstream media that has criticised these groups’ actions. Many of those within the wider anti-tourism industry movement itself have baulked at the scapegoating of tourists and the acts of violence and vandalism directed at foreign visitors to the city and tourism businesses such as hotels, restaurants and vehicle hire companies. For some, such acts constitute an act of opportunism by Arran as it jockeys along with the CUP to be seen as the main protagonist in the struggle against mass tourism. In my own case, by focusing on largely innocent tourists, such acts tend to shift attention away from the real structural and agential causes of overtourism in Barcelona, namely, a redevelopment model that prioritises profits over the people. In so doing, it plays into the hands of elite actors, who rather than seeing the action as an expression of legitimate concern, are given licence to dismiss it as evidence of the ‘tourismphobic’ attitudes of which the activists stand accused. 

 The only other criticism of the article relates to its use of evidence. Whereas many of the arguments in the paper are supported by appropriate evidence such as quotes from the media sources that make up the corpus, I would have liked this to go further. Thus, in some of the sections there is little in the way of evidence to substantiate the main claims. For example, in the section titled, ‘Press in the United Kingdom and Germany’, I would have welcomed the inclusion of quotes demonstrating the sort of ‘epidemiological narrative that expressed the fear of the anti-tourism protests spreading to other destinations’. Similarly, the section titled, ‘Statements in defense of tourism’ might also have benefitted from examples of media output underlying, ‘both the economic value in terms of the GDP’ of tourism and ‘its role in job creation'.   

 Finally, it is important to say that these criticisms in no way detract from what is an excellent study that contains real insight into the anti-tourism protests witnessed in Spain and the media’s response to them. For anyone with an interest in these issues, Araya López’ contribution is a worthy addition to the growing body of scholarship looking at this important issue. 

 Bibliography 

 Bruttomesso, E: Making sense of the square: Facing the touristification of public space through playful protest in Barcelona. Tourist Studies. 2018; 18(4), 467–485.  

 Cocola-Gant, A., & Pardo, D: Resistng tourism gentrifcaton: the experience of grassroots movements in Barcelona. Journal of Urban Design and Planning. 2017; May-August (13), 39-51. 

 Hughes N: ‘Tourists go home’: anti-tourism industry protest in Barcelona. Soc Mo Stud. 2018; 17(4): 471–477. Publisher Full Text 

 Hughes N, Mansilla JA: Political Discourse Analysis of the Degrowth Challenge to Dominant Tourism Narratives in Spain. In K. Andriotis (Ed.) Issues and Cases of Degrowth in Tourism. Wallingford: CABI: 2021; 86–103. Reference Source 

 Hughes, N: The Language of Protest: Graffiti slogans and the construction of tourism contestation in Barcelona in Ma, Y, Pothing, K & Rawnsley, G (eds) Elgar Handbook of Political Propaganda, in press, Cheltenham, Edward Elgar 

 Milano, C., Novelli, M., Cheer, J: Overtourism and Tourismphobia: A Journey Through Four Decades of Tourism Development, Planning and Local Concerns, Tourism Planning & Development. 2019: 16 (4), 353-357.

If applicable, is the statistical analysis and its interpretation appropriate?

Yes

Is the study design appropriate and is the work technically sound?

Yes

Is the work clearly and accurately presented and does it engage with the current literature?

Yes

Are the conclusions drawn adequately supported by the results?

Yes

Are sufficient details of methods and analysis provided to allow replication by others?

Yes

Are all the source data and materials underlying the results available?

Yes

Reviewer Expertise:

Online Learning, Critical Discourse Analysis, Spanish Political Studies

I confirm that I have read this submission and believe that I have an appropriate level of expertise to confirm that it is of an acceptable scientific standard.

## References

[1] : Making sense of the square: Facing the touristification of public space through playful protest in Barcelona. *Tourist Studies* .2018;18(4) : 10.1177/1468797618775219 467-485 10.1177/1468797618775219

[2] : Resisting tourism gentrification: the experience of grassroots movements in Barcelona. *Journal of Urban Design and Planning* .2017;39-51

[3] : ‘Tourists go home’: anti-tourism industry protest in Barcelona. *Social Movement Studies* .2018;17(4) : 10.1080/14742837.2018.1468244 471-477 10.1080/14742837.2018.1468244

[4] : Political Discourse Analysis of the Degrowth Challenge to Dominant Tourism Narratives in Spain. *Issues and Cases of Degrowth in Tourism* .2021;86-103

[5] : The Language of Protest: Graffiti slogans and the construction of tourism contestation in Barcelona. *Elgar Handbook of Political Propaganda* .in press;

[6] : Overtourism and Tourismphobia: A Journey Through Four Decades of Tourism Development, Planning and Local Concerns. *Tourism Planning* .

# Peer review: Reviewer response for version 1

**DOI:** 10.21956/openreseurope.14323.r27080

**Published:** 2021-06-22

**Authors:** Freya Higgins-Desbiolles

**Affiliations:** 1 School of Management, University of South Australia, Adelaide, Australia

This article conducted a discourse analysis of media reporting on public protests and activism organised by Arran in Spain in 2017, particularly focused on Barcelona. Situating the study in literature on media and journalism, social movement theory and tourism, this article explained how media reporting worked to delegitimise the protestors/protests and dilute the impact of their messages. The focus of this work is a consideration of how media discourse impacts the politics, power and decision-making associated with the development and management of tourism. It suggests that public protests and activism is a part of healthy democratic politics and in this case of Arran, has been delegitimised by media reporting at local, regional and international levels.

 The approach to discourse analysis was well explained and the data accessed; the analytic approach used provides confidence in the research methods.  Importantly, the author notes limitations to the research methods and indicates points for the reader to understand how the author arrived at the analysis offered. Additionally, a small section addressed the research ethics process as semi-structured qualitative interviews were undertaken.  These interviews were an important facet to this research as they provide a contrast to the media discourses studied and support the author’s argument that media reporting failed to accurately report on these acts of protest.

 As a result of the data analysis, the author identified four major media discourses: ‘tourismphobia’, politics, branding and the right to protest.  These themes are effectively explained, and the analysis really aids understanding of the dynamics occurring in the debates and concerns with mass tourism. The outcome of this explanation indicate that media reporting worked to present the Arran protests as radical, illegitimate, and akin to terrorism. Thus, rather than a neutral discourse on the contested nature of tourism in this community/ies, the reporting served the interests of status quo interests and failed to inform the public about the debates and issues accurately and effectively. I note the author has accomplished this section effectively providing sufficient facts, analysis and reference to literature to really provide effective and valuable information here.

 Content from the interview with a media spokesperson from Arran follows (although four interviews were undertaken in total). I found this section to be a great asset to the article and I commend the author for undertaking this aspect of the study. This section provides a contrast to the discourse on Arran offered by the media and indicates that the delegitimisation of the protests/protest movement does a disservice to debates on community futures in the face of tourism.

 The conclusion highlights three main points: the impacts of local and national politics in these processes; the use of the “protest” paradigm and a delegitimisation strategy in discourses on protests/protestors; and weak/biased reporting leading to inaccurate information on motives and actions of protests. The author closes with recommendations to media to give greater value to local activists’ insights and to better report on the complexity of such issues. It would be valuable if this work also concluded on the reiteration that social movements and public protests are legitimate parts of democratic political processes and counter monied and corporate power interests in the political arena. Effective and sustainable tourism policies and processes are more likely to occur when this fundamental point is realised. This work makes an important contribution to our understanding of these issues.

 My one concern with this work is about the clarity of the section “terrorist attacks”: “The terrorist attacks in Barcelona and Cambrils, which took place on the afternoon of August 17 ^th^, changed the debate about the protest acts”. My recommendation is that a few sentences are added explaining these events and explicitly making it clear that these events had nothing to do with Arran protests although media and others did connect them together as the article explains. The author might consider a chart or timeline outlining relevant events as an appendix which might assist readers in their understanding.

If applicable, is the statistical analysis and its interpretation appropriate?

Yes

Is the study design appropriate and is the work technically sound?

Yes

Is the work clearly and accurately presented and does it engage with the current literature?

Yes

Are the conclusions drawn adequately supported by the results?

Yes

Are sufficient details of methods and analysis provided to allow replication by others?

Yes

Are all the source data and materials underlying the results available?

Yes

Reviewer Expertise:

Tourism politics, policy, governance, impacts and community rights. Qualitative research methods.

I confirm that I have read this submission and believe that I have an appropriate level of expertise to confirm that it is of an acceptable scientific standard.
